# The transcription factor FoxM1 activates Nurr1 to promote intestinal regeneration after ischemia/reperfusion injury

**DOI:** 10.1038/s12276-019-0343-y

**Published:** 2019-11-08

**Authors:** Guo Zu, Jing Guo, Tingting Zhou, Ningwei Che, Baiying Liu, Dong Wang, Xiangwen Zhang

**Affiliations:** 1 Department of Gastroenterology Surgery, The Dalian Municipal Central Hospital Affiliated of Dalian Medical University, 116033 Dalian, China; 2 Institute of Integrative Medicine, Dalian Medical University, 116044 Dalian, China; 3 Department of Neurology, The First Affiliated Hospital of Dalian Medical University, 116011 Dalian, China; 4 Department of Neurosurgery, The Second Affiliated Hospital of Dalian Medical University, 116027 Dalian, China

**Keywords:** Trauma, RNAi

## Abstract

FoxM1 is involved in the regeneration of several organs after injury and expressed in the intestinal mucosa. The intrinsic mechanism of FoxM1 activity in the mucosa after intestinal ischemia/reperfusion (I/R) injury has not been reported. Therefore, we investigated the role of FoxM1 in mediating intestinal mucosa regeneration after I/R injury. Expression of FoxM1 and the proliferation of intestinal mucosa epithelial cells were examined in rats with intestinal I/R injury and an IEC-6 cell hypoxia/reperfusion (H/R) model. The effects of FoxM1 inhibition or activation on intestinal epithelial cell proliferation were measured. FoxM1 expression was consistent with the proliferation of intestinal epithelial cells in the intestinal mucosa after I/R injury. Inhibition of FoxM1 expression led to the downregulation of Ki-67 expression mediated by the inhibited expression of Nurr1, and FoxM1 overexpression promoted IEC-6 cell proliferation after H/R injury through activating Nurr1 expression. Furthermore, FoxM1 directly promoted the transcription of Nurr1 by directly binding the promoter of Nurr1. Further investigation showed low expression levels of FoxM1, Nurr1, and Ki-67 in the intestinal epithelium of patients with intestinal ischemic injury. FoxM1 acts as a critical regulator of intestinal regeneration after I/R injury by directly promoting the transcription of Nurr1. The FoxM1/Nurr1 signaling pathway represents a promising therapeutic target for intestinal I/R injury and related clinical diseases.

## Regenerative medicine: a drug target for intestinal injury

A signaling pathway that promotes the regeneration of intestinal cells in rats represents a promising therapeutic target for treating intestinal injury in humans. A team led by Guo Zu and Jing Guo from Dalian Medical University in China investigated the role of a regulatory protein called FoxM1 in repairing intestinal damage after a period of inadequate blood flow to the tissues of the gastrointestinal tract. They showed in rat models that FoxM1 promoted the proliferation of intestinal cells after injury by activating other proteins in a particular signaling pathway. Looking at tissue samples from five people who experienced intestinal injury as a result of restricted blood flow, the researchers detected low expression levels of FoxM1 and its downstream signaling intermediaries. Boosting the activity of those proteins could help promote healing and regeneration.

## Introduction

Intestinal ischemia/reperfusion (I/R) injury is a common pathophysiological process in many clinical settings that includes small bowel transplantation, hemorrhagic shock, and necrotizing enterocolitis^1,2^. It can cause severe intestinal mucosa damage that provokes intestinal mucosal barrier dysfunction. Once the intestinal epithelium, one of the most rapidly proliferating tissues in the body, is damaged, it activates regeneration programs to restore its mucosal barrier function^3^. The intrinsic mechanism of intestinal mucosa regeneration is not always sufficient to restore mucosal barrier function damaged by I/R injury, which is associated with significant morbidity and mortality. The pathophysiology of intestinal regeneration after I/R injury is complex and involves many signaling pathways^4–6^. Several signaling pathways are involved in the proliferation of intestinal epithelial cells after I/R injury^7^. However, the intrinsic mechanisms of intestinal epithelial cell proliferation after I/R injury are still not known.

As a typical transcription factor, FoxM1 belongs to the family of Forkhead box (Fox) proteins and is associated with cell proliferation. It is expressed in several embryonic tissues and the testes, thymus and intestinal crypts in adult mice^8–10^. In addition, FoxM1 is a key regulator of cell cycle progression and critical for the replication of DNA and mitosis^11–13^. Studies have shown that FoxM1 expression is reactivated after organ injury and that FoxM1 has pleiotropic roles during mouse liver regeneration after partial hepatectomy injury^14^. Ackermann reported that FoxM1 is required for the proliferation of preexisting beta cells after 60% partial pancreatectomy^15^. Ye et al. demonstrated that the expression of FoxM1 accelerates DNA replication and hepatocyte mitosis in the regenerating liver^16^. FoxM1, a key regulator of quiescence and self-renewal in hematopoietic stem cells, is mediated by control of Nurr1 expression^17^, and our previous research found that Nurr1 promotes intestinal mucosa epithelial cell proliferation after I/R injury by inhibiting p21 expression^18^. FoxM1, which is collectively considered a typical proliferation-associated transcription factor, is expressed in intestinal crypts. However, the effects of FoxM1 in regeneration of the intestinal mucosa after intestinal injury have not been examined.

Here, we propose that FoxM1 plays an important role in promoting intestinal mucosa regeneration after I/R injury. We determined that FoxM1 promotes intestinal mucosa epithelial cell proliferation via promoting the expression of Nurr1. Mechanistically, our findings demonstrate the direct transcriptional regulation of Nurr1 by FoxM1 in intestinal mucosa regeneration after I/R injury and that the FoxM1/Nurr1 pathway is involved in intestinal regeneration after I/R injury, providing new and potential therapies for intestinal I/R injury.

## Materials and methods

### Intestinal I/R injury model and tissue analysis

Male wild-type Sprague-Dawley rats weighing between 180 and 220 g were purchased from the Animal Center of Dalian Medical University. The animal studies were performed at Dalian Medical University. The intestinal I/R injury model was described in a previous study in rats^19^. Briefly, after anesthetization of the rats with an intraperitoneal injection of pentobarbital (40 mg/kg), the superior mesenteric artery (SMA) and collateral vessels were interrupted with atraumatic clips. After 1 h of ischemia, the atraumatic clips were removed to initiate reperfusion for 3, 6, 12, or 24 h. Ileum tissue samples (1 cm) were collected for the various experimental evaluations required for this study. Rats in the sham group underwent laparotomy without SMA and collateral vessel occlusion. Rats in the sham group did not exhibit changes in FoxM1 expression, and pentobarbital anesthesia did not influence FoxM1 expression (supplementary material 1).

To test the roles of FoxM1 in intestinal mucosa regeneration after I/R injury, we used the FoxM1 inhibitor thiostrepton (TST) to inhibit the expression of FoxM1^20,21^. Rats were randomly divided into 4 groups: the sham, sham+TST, I/R, and I/R+TST groups. The sham and I/R groups were treated as described above, and 50 mg/kg TST was given daily by intraperitoneal injection for two days before I/R surgery. After 1 h of intestinal ischemia followed by 6 h of reperfusion, ileum tissue samples were collected.

To determine whether Nurr1-mediated FoxM1 promotes intestinal regeneration after I/R injury, we determined whether the Nurr1 activator 1,1-bis(39-indolyl)-1-(p-chlorophenyl) methane (C-DIM12)^22,23^ could abolish the inhibitory effects of TST on intestinal I/R injury. Rats were randomly divided into 4 groups: the sham, I/R, I/R+TST, and I/R+TST+C-DIM12 groups. Rats in the sham, I/R and I/R+TST groups were treated as described above. C-DIM12 (50 mg/kg) was given before surgery. After 1 h of intestinal ischemia followed by 6 h of reperfusion, ileum tissue samples were collected.

The protocols and experiments in this study were conducted according to the guidelines of Dalian Municipal Central Hospital Affiliated of Dalian Medical University and approved by the Institutional Ethics Committee of Dalian Municipal Central Hospital Affiliated of Dalian Medical University.

### Western blot analysis

Protein expression in the intestinal samples or IEC-6 cells was analyzed by western blotting. The samples or cells were washed with ice-cold PBS and lysed in RIPA buffer (P0013, Beyotime, China) supplemented with phosphatase inhibitors and a protease inhibitor (P1048, Beyotime, China). Cells lysates were incubated for 2 h at 4 °C and centrifuged at 13,000 rpm for 10 min at 4 °C to remove cellular debris. The protein concentration was determined with a BCA protein assay kit (P0010, Beyotime, China). Whole cell lysates were loaded on a 12% SDS-polyacrylamide gel and transblotted to a nitrocellulose membrane after electrophoretic separation. Blocking was carried out in 5% PBS-milk, following which the membrane was incubated with anti-FoxM1 antibody (1:500, Santa Cruz Biotechnology, USA) and anti-Nurr1 antibody (1:500, Cell Signaling Technology, USA) overnight. The membrane was washed with 1× PBST and probed with HRP-conjugated anti-rabbit antibody (1:1000 dilution) for 2 h at 37 °C, followed by enhanced ECL chemiluminescence (P0018, Beyotime, China) detection. As a loading control, membranes were incubated with anti-β-actin antibody (TA-09, ZSGB-BIO, China) for 2 h at 37 °C, washed and probed with HRP-conjugated anti-mouse or anti-rabbit antibody. The results were calculated using the ratio of the density of the protein of interest corrected by the intensity of the protein used as the control (β-actin).

### Real-time PCR

Total RNA from intestinal tissues (in vivo) and IEC-6 cells (in vitro) was extracted. First-strand cDNA was synthesized, and target cDNA was amplified. The PCR primers were as follows: rat FoxM1 F: 5′-CAAGGTAAAAGCCACGTCTAAG C-3′, R: 5′-GGAGCAGCAGGTGACTAATGG-3′; rat Nurr1 F: 5′-CCAATCCGGC AATGACCAG-3′, R: 5′-TGATGATCTCCATAGAGCCAGTCAG-3′; rat β-actin F: 5′-CTGGAGAAGAGCTATGAGCTG-3′, R: 5′-AATCTCC TTCTGAT CCTGTC-3′; human FoxM1 F: 5′-GGAGGAAATGCCACACTTAGCG-3′, R: 5′-TAGGACTTCTTGGGTCTTGGGGTG-3′; human Nurr1 F: 5′-CATGGACCT CACCAACACTG-3′, R: 5′-AGTAAACCGACCCGGAGTG-3′; and human β-actin F: 5′-ACCCTGAAGTACCCCATCGAG-3′; R: 5′-ACATGATCTGGGTCATCTTCTCG-3′. mRNA expression was quantified using a TransStart Top Green qPCR SuperMix kit (TransGen Biotech, Beijing, China). β-Actin was used as an endogenous control, and the ΔΔCT method was used to analyze relative mRNA expression.

### Histological and immunohistochemical staining

Histological and immunohistochemical staining was performed on paraffin-embedded intestinal tissue. Immunohistochemical staining was performed using primary antibody against Ki-67, a nuclear protein associated with cell proliferation (ab15580, 1:200 dilution, Abcam, USA). Then, a streptavidin-biotin-peroxidase kit (ZSGB-BIO, Beijing, China) was used according to the manufacturer’s instructions. We measured the proliferation index, which was calculated as the average number of Ki-67-positive enterocytes per 100 enterocytes. At least 2000 enterocytes were assessed for these calculations^18^. Analysis and evaluation of the immunostaining results were independently carried out by two authors. Differences of opinion were reassessed together to reach a consensus.

### Cell culture and hypoxia/reoxygenation (H/R) model

IEC-6 cells were cultured, and an H/R model was developed as previously described^18^. IEC-6 cells were obtained from American Type Culture Collection (Manassas, VA, USA) and cultured at 37 °C in a 5% CO_2_ incubator. Dulbecco’s modified Eagle’s medium (DMEM; Gibco BRL) was used as the cell medium. IEC-6 cells were incubated under hypoxic conditions (5% CO_2_, 1% O_2_, and 94% N_2_).

### siRNA transfection

Sequence-specific siRNA was used for RNA interference in IEC-6 cells using Lipofectamine 2000 (Invitrogen, Shanghai, China). The siRNA sequences against FoxM1 were as follows: si-FoxM1: sense, 5′-GGAUGUGACUUACAUCGUUtt-3′; antisense, 5′-AACGAUGUAAGUCACAUCCtt-3′. The effects of each siRNA were analyzed using RT-PCR and western blotting.

### Cell proliferation assay

The proliferation of IEC-6 cells was assessed using a Cell Counting Kit-8 assay (Dojindo Molecular Technologies, Inc., Tokyo, Japan) according to the manufacturer’s recommendations. A 96-well plate was seeded with IEC-6 cells at a total density of 2 × 10^3^ cells/well in 100 μL of DMEM. Cells were allowed to attach and then subjected to H/R. At the indicated time points, the cells were washed with 100 μL of DMEM. The cells were incubated for 2 h in 10 μL of CCK-8 solution, and the absorbance at 450 nm was measured using an ELISA microplate reader (Thermo Scientific, USA).

### Plasmid construction and transient transfection

pEX-3 (pGCMV/MCS/Neo) plasmids expressing FoxM1 or Nurr1 were synthesized and purchased from GenePharma (Shanghai, China). The pEX-3 plasmid or control vector (2 µg) were used to transfect IEC-6 cells. After transfection using Lipofectamine 2000 for 48 h, G418 (400 µg/mL) was used for cell selection. Stably transduced IEC-6 cells were used for subsequent experiments.

### Chromatin immunoprecipitation (ChIP) assay

A ChIP assay kit (Beyotime, Shanghai, China) was used for ChIP assays performed as previously described^18^. Briefly, cells were treated with paraformaldehyde. Quenched cells were lysed, followed by sonication. A specific antibody (2 µg) was used for immunoprecipitation. DNA/protein complexes were eluted in elution buffer. Purified DNA was used for PCR analysis. PCR products were generated using the following primers: rat Nurr1 site 1: F, 5′-ATCCCATCTGGCTGACTTGT-3′, R, 5′-TCAGCAAATCTTA GCAACCGT-3′; Nurr1 site 2: F, 5′-GGGATTCCAGGGTGTGCTAT-3′, R, 5′-TCA GCAAATCTTAGCAACCGT-3′; and rat GAPDH: F, 5′-CGTAGCTCAGGCCTCTG CGCCCTT-3′, R, 5′-GCACTGCACAAGAAGAT GCGGCTG-3′.

### Luciferase reporter assay

The wild-type rat Nurr1 promoter and mutated Nurr1 promoter, that harbored a mutation in the FoxM1-binding motif were synthesized by GenePharma (Shanghai, China). The wild-type and mutated promoters were separately cloned into the pGL3-Basic luciferase vector between its NheI and HindIII sites. When IEC-6 cells reached 60–70% confluence, they were transfected with plasmid (2 µg/well) with Lipofectamine 2000. After transfection for 48 h, the cells were lysed with cell lysis buffer, and dual-luciferase reporter assay reagents (TransGen Biotech, Beijing, China) were then used according to the manufacturer’s instruments. Luciferase activities were determined and normalized to Renilla luciferase activity.

### Immunofluorescence

In vivo intestinal tissue samples were cryosectioned (10 µm thickness), and IEC-6 cells were postfixed in 4% paraformaldehyde in vitro. Then, tissues and cells were incubated with a primary antibody. The primary antibodies used were as follows: primary anti-Ki-67 (ab15580, 1:200 dilution, Abcam, USA) and primary anti-phosphorylated histone H3 (pH3, a mitosis marker) (1:50, Biogot Technology, Co., Ltd., Nanjing, China). Then, a secondary antibody (Invitrogen Life Technologies, Carlsbad, CA, USA) and DAPI (Beyotime, Shanghai, China) were added. A Leica DM 4000B microscope was used to examine staining. We also measured the proliferation index, which were the same as those of immunohistochemical staining, to analyze and evaluate proliferation of the intestinal mucosa.

### Patients

Five ischemic intestinal samples were collected from 5 clinical patients who underwent intestinal ischemia at Dalian Municipal Central Hospital Affiliated of Dalian Medical University. Written informed consent was obtained from the families of patients. A total of 5 patients underwent an operation for acute mesenteric arterial embolism, strangulated intestinal obstruction or incarcerated hernia. We excluded patients with tumors or inflammatory bowel disease, patients who took immunosuppressants and patients who disagreed. The patients consisted of four males and one female with an average age of 66.6 years. The ischemia duration for the intestinal samples was greater than 6 h. After excision of ischemic intestinal tissue, small intestine tissue samples were harvested and stored immediately in liquid nitrogen for western blotting and PCR analysis. Tissues from another part of the intestine were fixed in 10% formalin for histological and immunohistochemical staining. Intestinal samples were obtained with the approval of the Institutional Ethical Committees of Dalian Municipal Central Hospital Affiliated of Dalian Medical University.

### Statistical analysis

All values are expressed as the means ± SDs. Analysis of data between two groups was performed using two-tailed Student’s *t*-tests. One-way analysis of variance and Student-Newman-Keuls tests were used to compare means among more than two groups. All data analyses were performed with Statistical Product and Service Solutions and GraphPad Prism 5.0. *P* < 0.05 indicated significance.

## Results

### FoxM1 is induced during intestinal mucosa regeneration after I/R

**Fig. 1:**
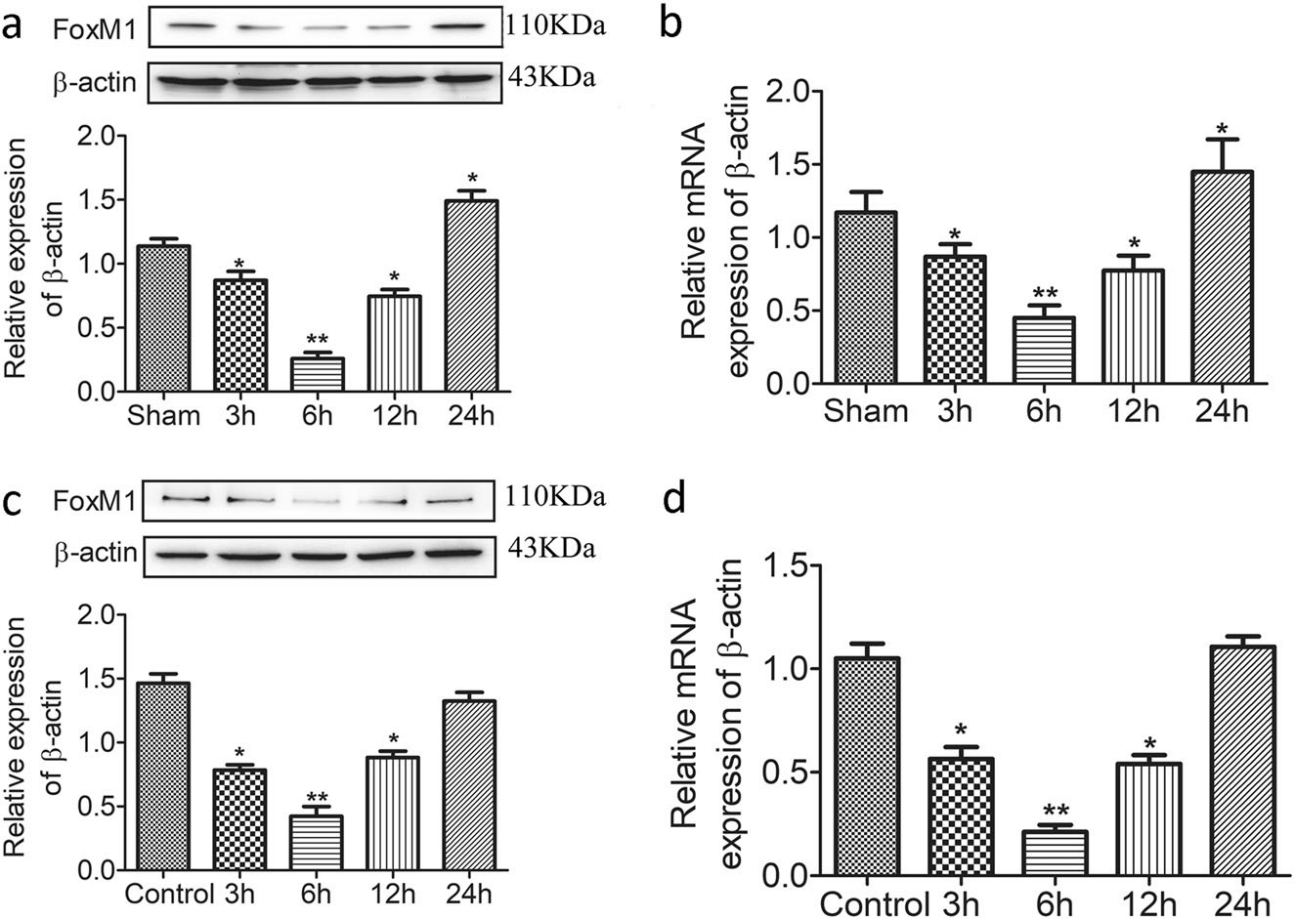
FoxM1 is induced during intestinal regeneration after I/R in vivo and in vitro. **a**, **b** Representative protein and mRNA levels of FoxM1 in rats subjected to ischemia for 1 h followed by 3, 6, 12, or 24 h of reperfusion (*n* = 6). **c**, **d** Representative protein and mRNA levels of FoxM1 in IEC-6 cells subjected to 6 h of hypoxia followed by 3, 6, 12, or 24 h of reoxygenation (*n* = 6). The values are presented as the means ± SDs. ^*^P < 0.05 compared to the sham group or control group, ^**^P < 0.01 compared to the sham group or control group

We previously investigated intestinal epithelium regeneration after 1 h of ischemia and 3, 6, 12, or 24 h of reperfusion^18^. To determine the expression of FoxM1 in intestinal mucosa regeneration after I/R injury, we tested the expression of FoxM1 in intestinal tissues that underwent 1 h of ischemia followed by 3, 6, 12, or 24 h of reperfusion. The protein and mRNA expression levels of FoxM1 were tested after reperfusion for different lengths of time (Fig. 1a, b). The mRNA and protein expression levels of FoxM1 were consistent with proliferation of the intestinal epithelium after I/R injury. These results indicated that FoxM1 is induced during intestinal mucosa regeneration after I/R injury in vivo.

We also examined FoxM1 expression in IEC-6 cells in vitro. After 6 h of hypoxia and 3, 6, 12, or 24 h of reoxygenation, the expression of FoxM1 was associated with IEC-6 cell proliferation after I/R injury (Fig. 1c, d). Overall, these findings suggested that FoxM1 is induced during the proliferation of IEC-6 cells after H/R in vitro.

### Reduced expression of FoxM1 inhibits intestinal epithelial proliferation after I/R in vivo

**Fig. 2:**
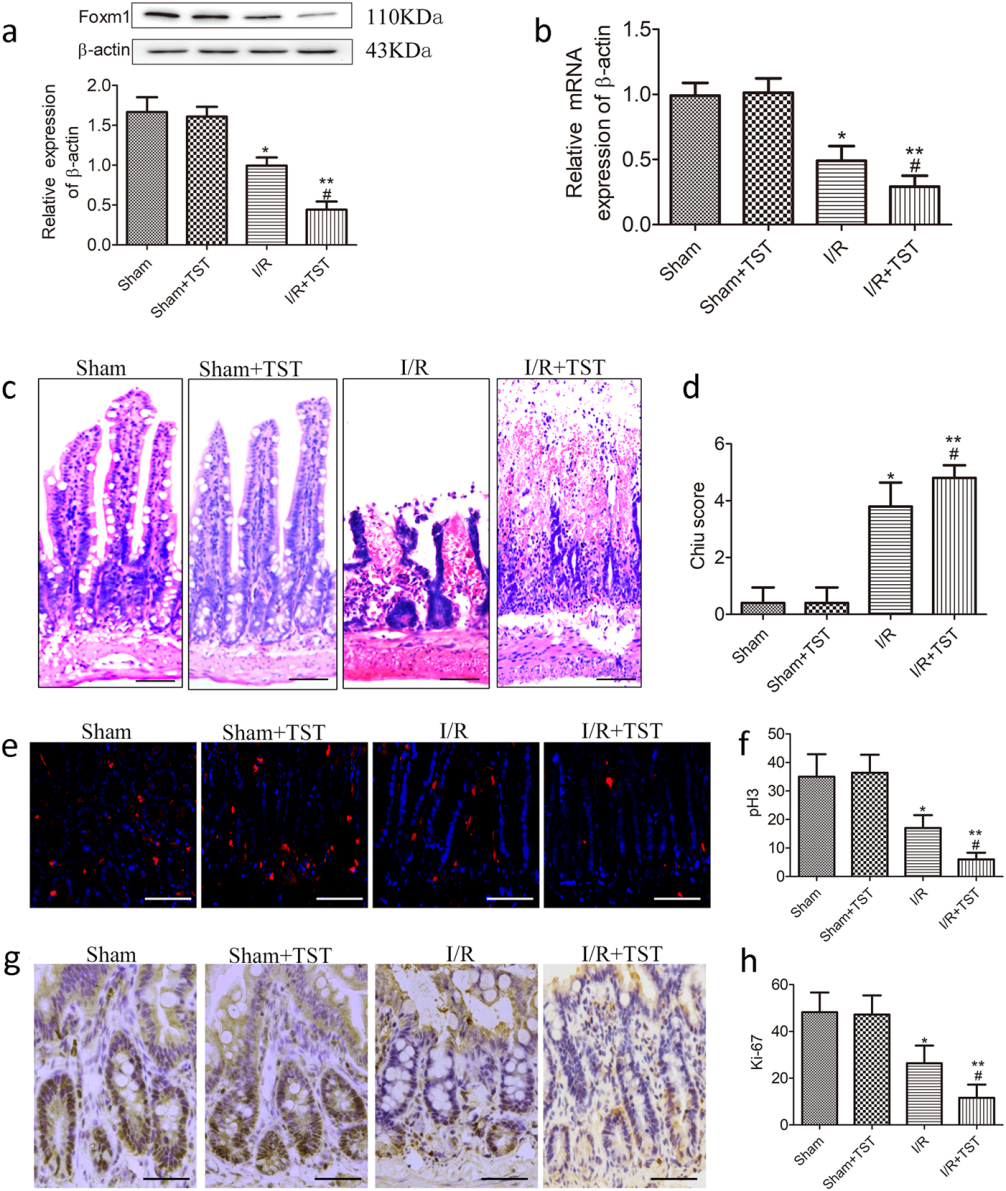
Reduced expression of FoxM1 by TST inhibits intestinal epithelial proliferation after I/R in vivo. **a** Representative protein levels of FoxM1 in the intestinal tissues of different groups (*n* = 6). **b** Representative mRNA levels of FoxM1 in the intestinal tissues of different groups (*n* = 6). **c**, **d** HE staining and Chiu scores of intestinal tissues (*n* = 5) (bar = 50 µm). **e**, **f** Immunofluorescence staining for pH3 in the intestinal tissues of different groups (*n* = 5) (bar = 100 µm). **g**, **h** Immunohistochemical staining for Ki-67 (*n* = 5) (bar = 100 µm). The values are presented as the means ± SDs. **P < 0.01 compared to the sham group, ^#^*P* < 0.05 compared to the I/R group, ^##^*P* < 0.01 compared to the I/R group

As FoxM1 was induced during mucosa regeneration after intestinal I/R injury, we used the FoxM1 inhibitor TST to test the effect of FoxM1 on intestinal epithelial proliferation^20,24^. First, the data suggested that TST inhibited the expression of FoxM1 (Fig. 2a, b). Then, we investigated the effects of TST on intestinal epithelial proliferation and histology after I/R injury. The I/R group showed impaired intestinal villi and reduced proliferation in the intestinal epithelium compared with the sham group (Fig. 2c, d). The group treated with TST following intestinal I/R injury showed worsened injury and an increased Chiu score compared to the I/R group. In addition, proliferation in the intestinal epithelium after I/R injury was further reduced (Fig. 2e, h). Collectively, these findings suggested that the reduced expression of FoxM1 inhibits intestinal epithelial proliferation induced by intestinal I/R.

### FoxM1 promotes IEC-6 cell proliferation after H/R injury

**Fig. 3:**
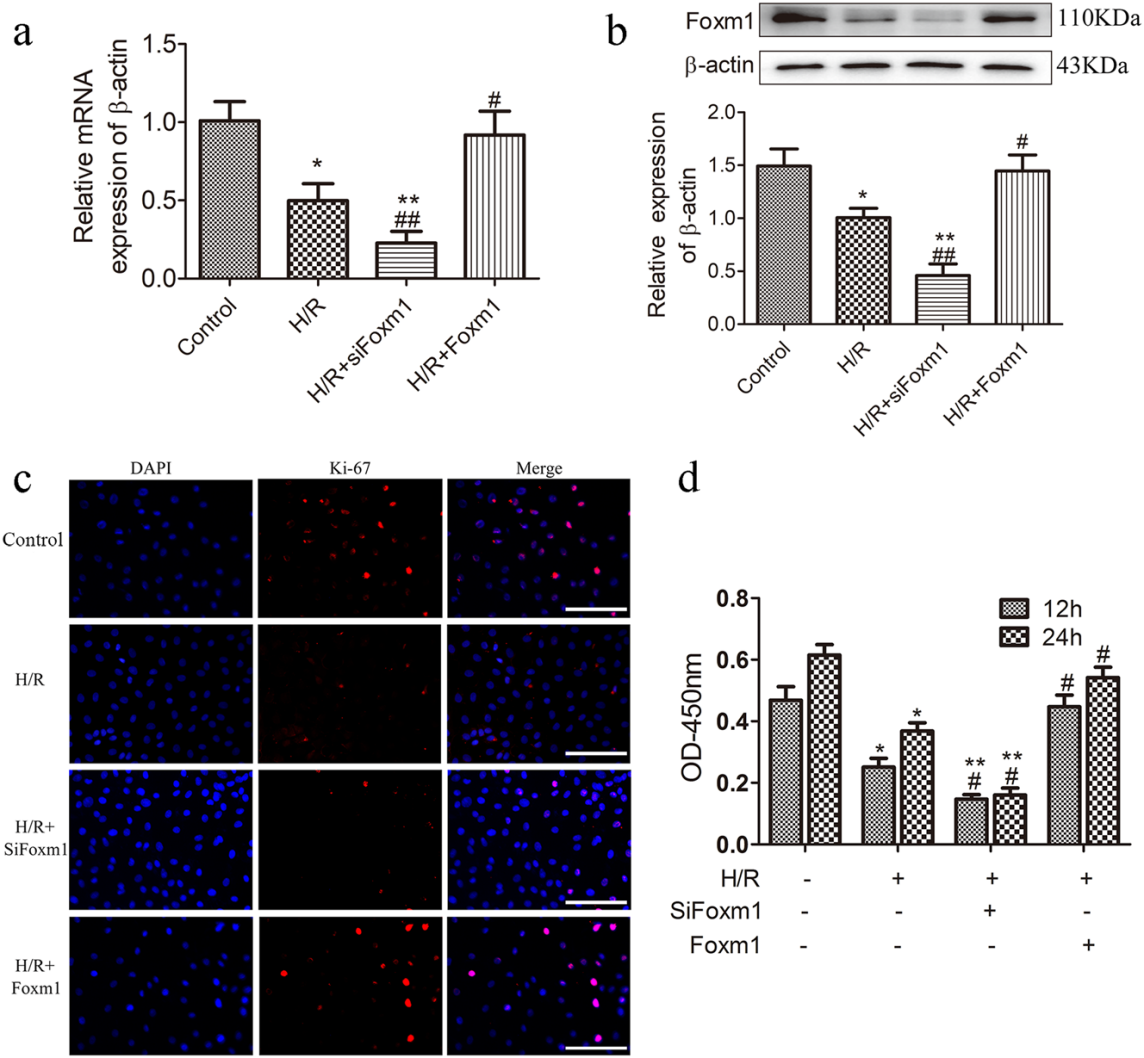
FoxM1 promotes IEC-6 cell proliferation after H/R injury. **a** Representative mRNA levels of FoxM1 in IEC-6 cells in different groups (*n* = 6). **b** Representative protein levels of FoxM1 in IEC-6 cells in different groups (*n* = 6). **c** Immunofluorescence staining for pH3 in IEC-6 cells in different groups (*n* = 5) (bar = 100 µm). **d** The CCK-8 assay was used to examine the cell proliferation of IEC-6 cells in different groups after hypoxia for 6 h and 12 h or 24 h of reoxygenation (*n* = 6). The values are presented as the means ± SDs. *P < 0.05 compared to the control group, **P < 0.01 compared to the control group, ^#^*P* < 0.05 compared to the I/R group, ^##^*P* < 0.01 compared to the I/R group

As reduced expression of FoxM1 inhibited intestinal epithelial proliferation after I/R, we next tested the effect of FoxM1 knockdown and overexpression on IEC-6 cell proliferation after H/R injury. FoxM1 knockdown using siRNA (siFoxM1), FoxM1 overexpression using plasmid transfection (Fig. 3a, b) and IEC-6 cell proliferation after 12 h and 24 h of reoxygenation following 6 h of hypoxia were tested by the CCK-8 assay and determining the expression of Ki-67. FoxM1 knockdown dramatically decreased the proliferation of IEC-6 cells compared with that in the H/R group, whereas FoxM1 overexpression increased the proliferation of IEC-6 cells compared with that in the H/R group (Fig. 3c, d). These results showed that FoxM1 promotes the proliferation of IEC-6 cells after H/R injury.

### Nurr1 is induced by FoxM1 during intestinal regeneration after I/R injury in vivo and in vitro

**Fig. 4:**
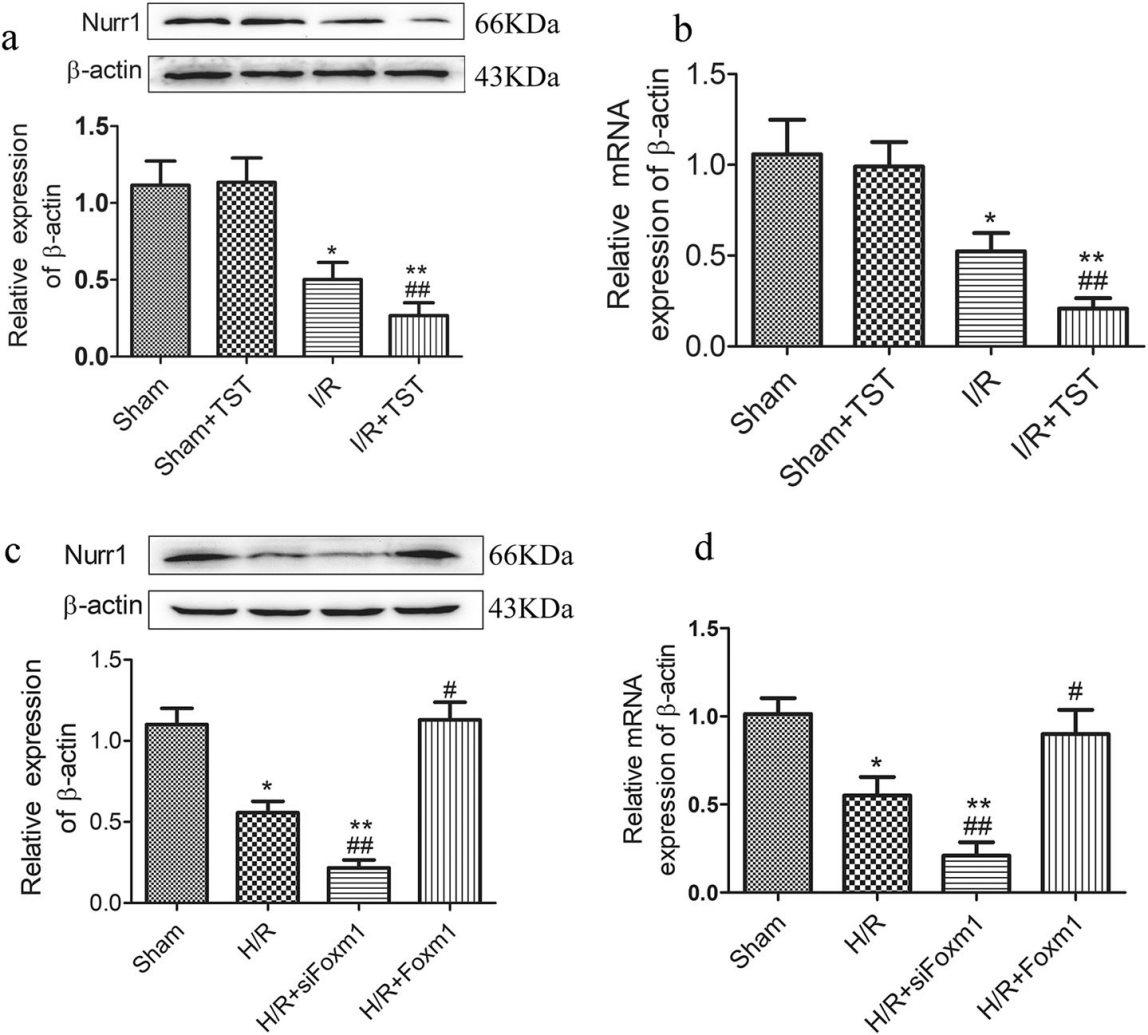
Nurr1 is induced by FoxM1 intestinal regeneration after I/R in vivo and in vitro. **a** Representative protein levels of Nurr1 in the rats of different groups determined by western blotting (*n* = 6). **b** Representative mRNA levels of Nurr1 in the rats of different groups determined by real-time PCR (*n* = 6). **c** Representative protein levels of Nurr1 in IEC-6 cells of different groups determined by western blotting (*n* = 6). **d** Representative mRNA levels of Nurr1 in IEC-6 cells in different groups determined by real-time PCR (*n* = 6). The values are presented as the means ± SDs. ^*^P < 0.05 compared to the sham group, ^**^P < 0.01 compared to the sham group, ^#^*P* < 0.05 compared to the I/R group, ^##^*P* < 0.01 compared to the I/R group

As a transcriptional factor, Nurr1 promotes intestinal mucosa regeneration after I/R injury^23^. Hou reported that FoxM1 can regulate Nurr1 expression in mouse and human leukemia cells^17^. Thus, we examined whether FoxM1-mediated intestinal epithelial proliferation is mediated by Nurr1. As expected, Nurr1 and FoxM1 expression levels were similarly inhibited in intestinal tissues after I/R injury (Fig. 4a, b). Similar results were demonstrated following the knockdown and overexpression of FoxM1 in IEC-6 cells after H/R injury (Fig. 4c, d). These results indicated that Nurr1 is induced by FoxM1 during intestinal regeneration after I/R injury in vivo and in vitro.

### FoxM1 promotes intestinal regeneration after I/R injury in a Nurr1-dependent manner

**Fig. 5:**
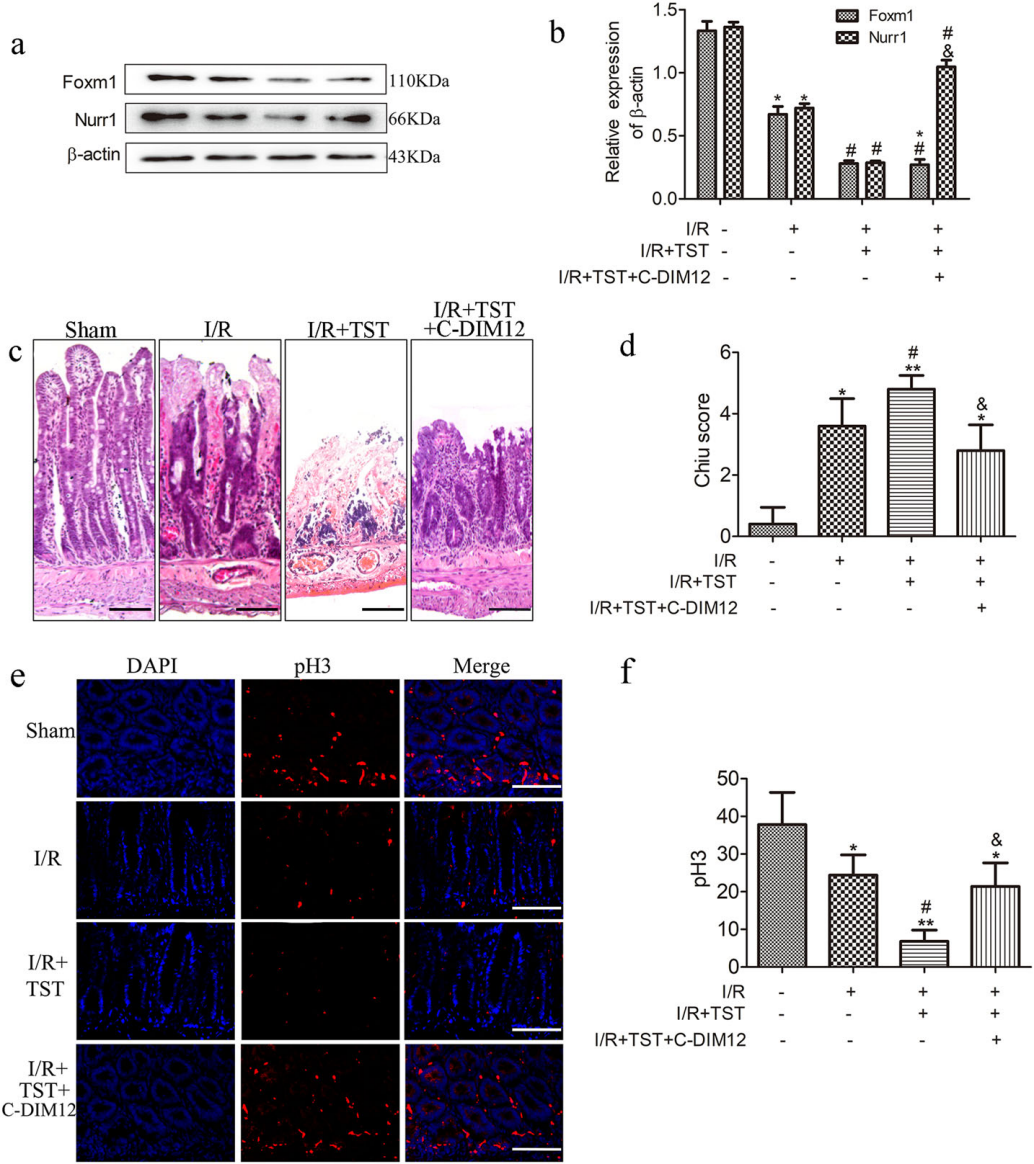
Nurr1 mediates FoxM1 and promotes intestinal regeneration after I/R injury. **a**, **b** Representative protein levels of FoxM1 in the intestinal tissues of different groups (*n* = 6). **c**, **d** HE staining and Chiu scores of intestinal tissues (*n* = 5) (bar = 50 µm). **e**, **f** Immunofluorescence staining for pH3 in the intestinal tissues of different groups (*n* = 5) (bar = 50 µm). The values are presented as the means ± SDs. *P < 0.05 compared to the sham group, **P < 0.01 compared to the sham group, ^#^*P* < 0.05 compared to the I/R group, ^&^*P* < 0.05 compared to the I/R+TST group

As Nurr1 expression is closely associated with FoxM1 activation, we hypothesized that FoxM1 promotes intestinal regeneration after I/R injury in a Nurr1-dependent manner. We used the Nurr1 activator C-DIM12 to examine the role of Nurr1 in the inhibition of FoxM1. Compared with the I/R group, the group treated with the FoxM1 inhibitor exhibited decreased FoxM1 expression (Fig. 5a, b). Furthermore, the FoxM1 inhibitor obviously inhibited proliferation of the intestinal epithelium after I/R injury. Nurr1 expression in the intestine was activated with the FoxM1 inhibitor TST, and TST no longer exerted any inhibitory effects of proliferation of the intestinal epithelium after I/R injury (Fig. 5c–f). These results showed that FoxM1 promotes intestinal regeneration after I/R injury in a Nurr1-dependent manner.

### FoxM1 promotes IEC-6 cell proliferation after H/R injury in a Nurr1-dependent manner

**Fig. 6:**
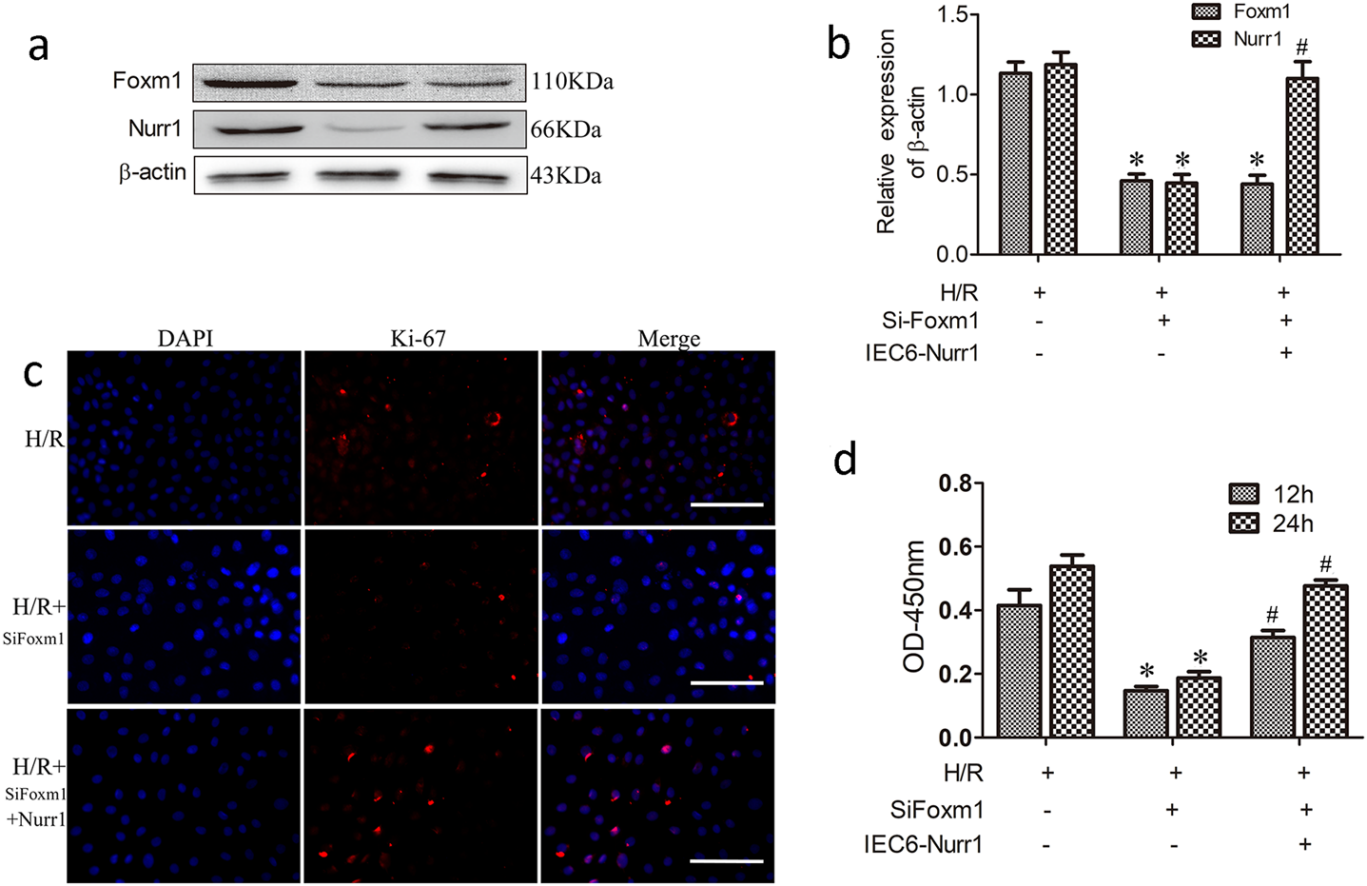
FoxM1 promotes IEC-6 cell proliferation after H/R injury in a Nurr1-dependent manner. **a**, **b** Representative protein levels of FoxM1 in IEC-6 cells in different groups (*n* = 6). **c** Immunofluorescence staining for Ki-67 in IEC-6 cells in different groups (*n* = 5) (bar = 100 µm). **d** The CCK-8 assay was used to examine the cell proliferation of IEC-6 cells in different groups (*n* = 6). The values are presented as the means ± SDs. *P < 0.05 compared to the H/R group, ^#^*P* < 0.05 compared to the H/R + siFoxM1 group

Our results have showed that FoxM1 promotes intestinal regeneration after I/R injury in a Nurr1-dependent manner in vivo. We further detected the effect of Nurr1 overexpression in proliferation of IEC-6 cells with FoxM1 knockdown after H/R injury. The results showed that Nurr1 was overexpressed in IEC-6 cells with FoxM1 knockdown by plasmid transfection (Fig. 6a, b). Overexpression of Nurr1 reversed the inhibition of IEC-6 cell proliferation induced by FoxM1 knockdown (Fig. 6c, d). These results suggest that FoxM1 promotes IEC-6 cell proliferation after H/R injury in a Nurr1-dependent manner.

### FoxM1 regulates Nurr1 expression by directly inhibiting Nurr1 expression

**Fig. 7:**
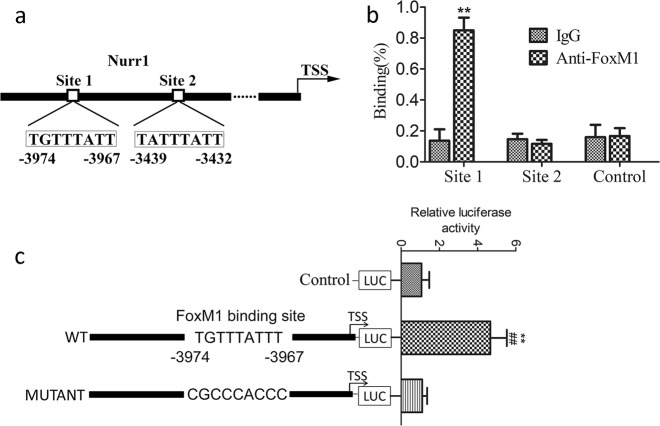
FoxM1 regulates Nurr1 expression by directly inhibiting Nurr1 expression. **a** Schematic diagram of the wild-type (WT) rat Nurr1 upstream promoter region showing the predicted FoxM1-binding regions: site 1 and site 2. TSS indicates the transcription start site. **b** Endogenous binding of FoxM1 to the upstream region of Nurr1 in IEC-6 cells after H/R injury as determined by ChIP assay. IgG was used as a negative control. **c** The TGTTTATTT core motif was mutated into CGCCCACCC in the mutant Nurr1 promoter luciferase construct. Luciferase reporter assays: IEC-6 cells were transfected with wild-type or mutated Nurr1 promoter luciferase constructs and either FoxM1 or pEX-3 vectors

Our previous results showed that FoxM1 promotes intestinal mucosa regeneration after I/R injury in a Nurr1-dependent manner. In addition, Hou reported that FoxM1 can directly regulate Nurr1 transcription in mice. To test the relationship between FoxM1 and Nurr1 in rats, we searched the proximal region of the Nurr1 promoter for the consensus FoxM1-binding site [T(G/A)TTT(G/A)TT]^25^. Two putative FoxM1-binding sites were identified upstream of the Nurr1 transcription start site (TSS) (Fig. 7a). In addition, a ChIP assay using IEC-6 cells showed that FoxM1 bound to site 1 of the upstream region of Nurr1 TSS directly, but not site 2 (Fig. 7b). Next, we performed dual-luciferase reporter assays to determine whether FoxM1 binds site 1 of the Nurr1 promoter. IEC-6 cells were cultured and transfected with plasmid containing wild-type or site 1 mutant Nurr1 promoter. Luciferase activity of cells expressing the wild-type Nurr1 promoter containing binding site 1 was activated by FoxM1 expression (Fig. 7c). These results revealed that the integrity of binding site 1 upstream of the Nurr1 TSS is required for FoxM1-mediated activation of Nurr1 expression.

### Downregulation of FoxM1/Nurr1 in the ischemic intestine of clinical patients

**Fig. 8:**
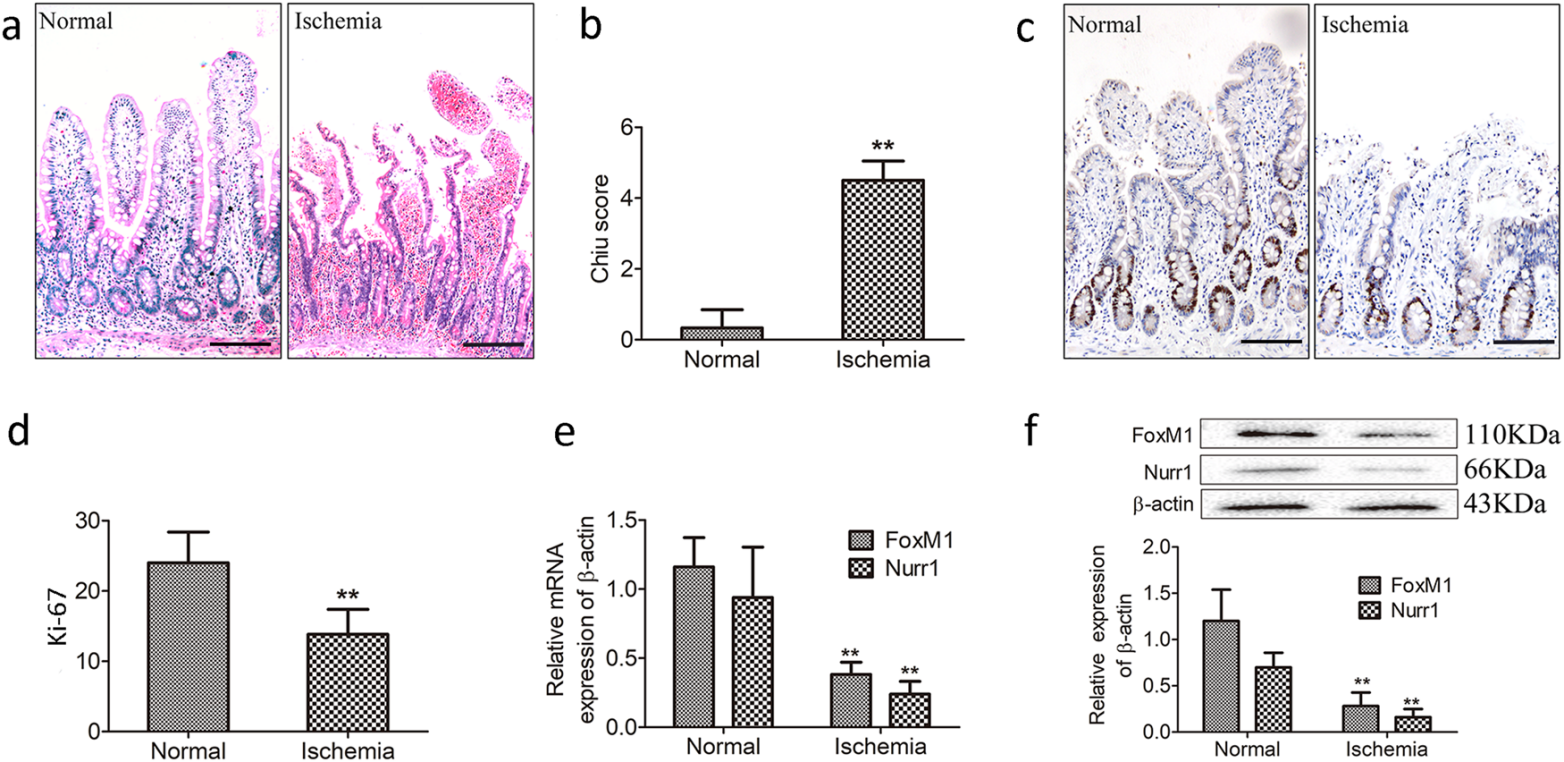
Downregulation of FoxM1/Nurr1 in the ischemic intestine of clinical patients. **a**, **b** Histology and Chiu scores of ischemic intestinal tissues from clinical patients (bar = 50 µm). **c**, **d** Immunohistochemical staining for Ki-67 (bar = 50 µm). **e** Representative mRNA levels of FoxM1 and Nurr1 (*n* = 5). **f** Representative protein levels of FoxM1 and Nurr1 (*n* = 5). The values are presented as the means ± SDs. **P < 0.01 compared to normal intestinal tissue

To determine the function of FoxM1 in the proliferation of intestinal epithelial cells of the human ischemic intestine, we investigated proliferation in the human ischemic intestine by HE (Fig. 8a, b) and immunohistochemical (Fig. 8c, d) staining and the expression of FoxM1 and Nurr1 in clinical patients with intestinal ischemia (Fig. 8e, f). We observed the decreased expression of FoxM1 and Nurr1 and the expression of Ki-67 in the human ischemic intestine. These results were similar to those in the rat model of intestinal I/R injury.

## Discussion

Intestinal I/R injury, a serious condition in intensive care units and among vascular surgery patients, is characterized by mucosal barrier damage. Once intestinal mucosal damage occurs, new intestinal crypt epithelial cells proliferate and migrate to the villus and subsequently restore proper mucosal barrier function^26,27^. Previous studies have shown that several genes are activated during the proliferation of intestinal epithelial cells after injury^28,29^. However, the intrinsic mechanism of intestinal epithelial proliferation remains unknown. Our previous study showed that Nurr1 is involved in intestinal regeneration after I/R injury by directly inhibiting p21 expression^23^. Our study is the first to find that (1) FoxM1 can be induced during intestinal mucosa regeneration after I/R injury, (2) FoxM1 promotes intestinal epithelial cell proliferation by promoting Nurr1 gene expression, and (3) Nurr1 is a novel downstream effector of FoxM1 that can be directly activated by FoxM1 and mediate the ability of FoxM1 to promote intestinal regeneration after I/R injury.

Intestinal epithelial cells proliferate to rebuild the proper structure of the epithelium after I/R injury, and nuclear factors are involved in intestinal mucosa regeneration after injury^30–32^. FoxM1 is involved in the regeneration of several organs after injury^33,34^. As a nuclear factor, FoxM1 promotes organ regeneration after injury via its transcriptional regulation of downstream genes^35,36^. FoxM1 is highly expressed in intestinal crypts, where it stimulates proliferation by promoting cell cycle entry into S phase and M phase, and may be a key target for regeneration after injury to some organs. In addition, inflammation can promote tissue regeneration after injury through poorly understood mechanisms^37^. As a transcription factor, FoxM1 acts as a critical mediator of the inflammatory response. Zeng et al. revealed that the inhibition of FoxM1 suppresses the production of inflammatory factors including tumor necrosis factor-α and IL-6 in osteoarthritis. The inflammatory mediators nitric oxide, prostaglandin E2 and cyclooxygenase-2 were also repressed by FoxM1 knockdown^38^. It has been reported that inflammatory diseases drive tissue hypoxia^39,40^. FoxM1 is involved in tissue regeneration after injury^33,34^. In our study, FoxM1 was induced in intestinal regeneration after I/R injury. In addition, the expression of FoxM1 was consistent with that of Ki-67 and pH3 in the intestinal mucosa after I/R injury. These results revealed that the expression of FoxM1 is closely associated with intestinal epithelial cell proliferation. Furthermore, inhibition of the expression of FoxM1 resulted in the reduced proliferation of intestinal epithelial cells, and the activation of FoxM1 promoted increased cell proliferation. These data suggested that FoxM1 promotes intestinal mucosa regeneration after I/R injury.

Previous studies have shown that FoxM1 induces the expression of proliferation-associated genes that regulate cell cycle progression and promote cell proliferation in various tissues. Zhao et al. reported that overexpression of FoxM1 induces the expression of cell cycle-associated genes and promotes the proliferation of lung endothelial cells and epithelial cells in a different model of inflammatory lung injury^41,42^. Hou et al. reported that E2F1 target genes and other genes that promote S phase transition were increased when FoxM1 was absent in hematopoietic stem cells, providing molecular evidence of the promotion of S phase progression in hematopoietic stem cells and increased proliferation of hematopoietic stem cells. Moreover, downregulation of FoxM1 led to the loss of Nurr1^17^. Interestingly, our previous studies showed that the loss of Nurr1 markedly impairs intestinal epithelial cell proliferation and is associated with the upregulation of p21^18^. In this study, we found that the ectopic expression of Nurr1 could reverse the inhibition of intestinal epithelial cell proliferation induced by FoxM1 deletion, indicating that these roles are likely mediated by Nurr1. Furthermore, for the first time, our results demonstrated the physiological significance of the overexpression of FoxM1 and resultant epithelial regeneration in the mechanism of intestinal epithelial cell repair following I/R injury. FoxM1 activates Nurr1 expression, and the forced expression of Nurr1 reverses the inhibitory effect of FoxM1 deficiency on the proliferation of intestinal epithelial cells. Taken together, these results suggest that FoxM1 regulates intestinal epithelial cell proliferation through activating Nurr1-mediated pathways.

Next, we examined the intrinsic molecular mechanism by which FoxM1 inhibits the expression of Nurr1. FoxM1 can bind to and transactivate target promoters by recognizing and binding to a specific FoxM1-binding site. Hou et al. identified two putative FoxM1-binding sites upstream of the Nurr1 TSS (-5585 to -5576 and -167 to -159) and showed that consensus site 1 (-5585 to -5576) in the promoter of Nurr1 was required for FoxM1-mediated activation of Nurr1 expression (-5585 to -5576) in a mouse model^17^. Using bioinformatic analysis, we found a consensus FoxM1-binding site in the Nurr1 promoter that interacted with FoxM1. The results of subsequent ChIP and luciferase assays showed that the binding of FoxM1 to this site activated Nurr1 transcription. This evidence revealed the direct promotion of Nurr1 expression at the transcriptional level by FoxM1. However, other transcriptional factors that involve additional regulatory mechanisms cannot be excluded.

The role of FoxM1 in intestinal I/R injury is unknown. By analyzing the expression of FoxM1 in clinical patients with superior mesenteric artery embolus, we found that FoxM1 was significantly downregulated in the ischemic mucosa of intestinal I/R patients. In our previous study, we tested Nurr1 expression was downregulated in the intestinal mucosa after ischemic injury^18^. More importantly, the expression of FoxM1 and Nurr1 was reduced along with the expression of Ki-67 in the ischemic intestinal mucosa. Thus, our data implicated the critical role of FoxM1 in promoting human intestinal mucosa epithelial cells and suggested that the upregulation of Nurr1 expression contributes to the proliferation of epithelial cells of the human intestinal mucosa.

In summary, we show that FoxM1 acts as a critical regulator of intestinal mucosa regeneration after I/R injury by promoting the expression of Nurr1. Moreover, we revealed that FoxM1 promotes intestinal mucosa epithelial cell proliferation by directly binding the promoter of Nurr1 and promoting the transcription of Nurr1. Together, the results of our study reveal the important role of the FoxM1/Nurr1 signaling pathway in promoting intestinal regeneration after I/R injury. Therefore, we believe that the FoxM1/Nurr1 signaling pathway is a novel therapeutic target for intestinal I/R injury and related clinical diseases.

## Supplementary information

Supplemental figure 1 Legend

Supplemental material
