# Supplementary figures and images for: The transcription factor FoxM1 activates Nurr1 to promote intestinal regeneration after ischemia/reperfusion injury
Source: Exp Mol Med. 2019 Nov 8;51(11):132. doi: 10.1038/s12276-019-0343-y (PMC6841953; doi:10.1038/s12276-019-0343-y)

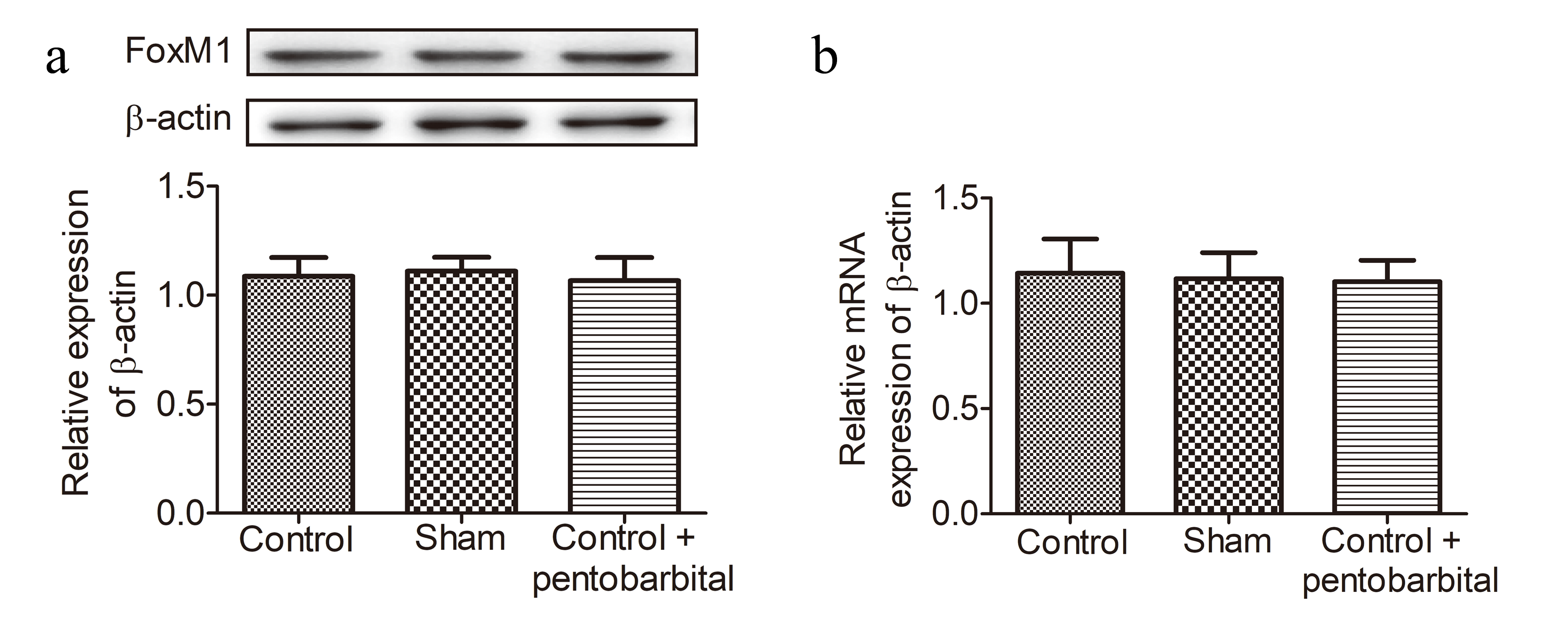

Supplement: Supplementary file 1 — Supplemental figure 1 Legend [file 12276_2019_343_MOESM1_ESM.tif]
