# Supplementary material for: The transcription factor FoxM1 activates Nurr1 to promote intestinal regeneration after ischemia/reperfusion injury
Source: Exp Mol Med. 2019 Nov 8;51(11):132. doi: 10.1038/s12276-019-0343-y (PMC6841953; doi:10.1038/s12276-019-0343-y)
Supplement: Supplementary file 2 — Supplemental material [file 12276_2019_343_MOESM2_ESM.docx]

**Supplemental material fig. 1 FoxM1 expression in intestinal tissue of control group, sham operation group and control + pentobarbital group.** (a) Representative protein levels of FoxM1 in the intestinal tissues of rats in the different groups (n =6). (b) Representative mRNA levels of FoxM1 in the intestinal tissues of rats in the different groups (n =6). The values are presented as means ± SDs.
